# Biopsychosocial Factors during the Perinatal Period: Risks, Preventative Factors, and Implications for Healthcare Professionals

**DOI:** 10.3390/ijerph18158206

**Published:** 2021-08-03

**Authors:** Ashley J. Blount, Charmayne R. Adams, Ann L. Anderson-Berry, Corrine Hanson, Kara Schneider, Gurudutt Pendyala

**Affiliations:** 1 Department of Counseling, University of Nebraska Omaha, Omaha, NE 68182, USA; charmayneadams@unomaha.edu (C.R.A.); karaschneider@unomaha.edu (K.S.); 2 Department of Pediatrics, University of Nebraska Medical Center, Omaha, NE 68198, USA; alanders@unmc.edu; 3 Department of Anesthesiology, University of Nebraska Medical Center Omaha, Omaha, NE 68198, USA; gpendyala@unmc.edu; 4 Medical Nutrition Education Division, University of Nebraska Medical Center, Omaha, NE 68198, USA; ckhanson@unmc.edu; 5 Child Health Research Institute, University of Nebraska Medical Center, Omaha, NE 68198, USA

**Keywords:** perinatal health, perinatal mental health, risk factors, biopsychosocial, perinatal health disparities

## Abstract

Women face risks to their wellbeing during the perinatal period of pregnancy. However, there is a dearth of information on perinatal risk factors within the biopsychosocial paradigm. Emphasis is often placed on biological components associated with pregnancy and women’s health. However, psychological and social determinants of health are integral during the perinatal period, and mental wellness is often a determinant for positive maternal and neonatal health outcomes. This article reviews risk factors of perinatal wellness (e.g., physical and nutritional concerns, trauma, discrimination, adverse childhood events) and highlights protective factors for women in their perinatal period. Healthcare professionals can support perinatal health by focusing on culturally and contextually appropriate research and prevention, providing equal access to sexual and reproductive healthcare information and services, providing quality education and training for helping professionals, and supporting policies for positive sexual and reproductive women’s healthcare.

## 1. Introduction

**Figure 1:**
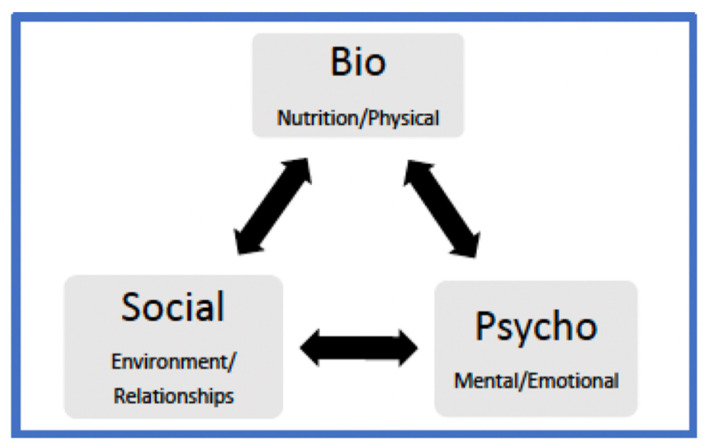
Example of Biopsychosocial Theoretical Pregnancy Model Incorporating Biological, Psychological, and Social Determinants of Health.

When focusing on the biopsychosocial risk factors related to perinatal health, it is important to consider a number of salient factors. Emphasis is placed on the biological components associated with pregnancy and women’s health, yet psychological and social determinants of health are integral during the perinatal period and are often determinants for positive maternal and neonatal health outcomes [1]. Thus, we attempt to bridge this gap by highlighting and emphasizing the importance of psychological and social determinants of perinatal health, along with biological factors. The World Health Organization (WHO) stated that “health is a state of complete physical, mental, and social well-being and not merely in the absence of disease or infirmity” [2] (p. 984). Additionally, viewing women’s health holistically allows healthcare professionals to account for a variety of factors influencing maternal wellbeing. As such, utilizing a biopsychosocial model to assess risk factors and protective factors during the perinatal period is fitting and can result in enhanced maternal and neonatal outcomes [3,4]. See Figure 1 for sample biopsychosocial pregnancy model.

In the biopsychosocial realm, risks contributing the health and wellness of perinatal women exist. Examples such as epigenetic changes, transgenerational trauma, mental illness, stress experiences, racism, and microaggressions contribute directly to health disparities and poor maternal and neonatal outcomes [5]. Healthcare professionals need to account for impacts of these risks on perinatal health and wellbeing. The following sections highlight biological, psychological, and social risk factors of the perinatal period through a thematic review of the literature on perinatal wellbeing. Search terms included perinatal, wellbeing, wellness, preventative factors, biopsychosocial (biological, psychological, social), and risk factors across medical and alternative medicine-based journals (e.g., counseling, holistic practices). Limitations of the literature review and implications for healthcare professionals working with and supporting women in their pregnancy lifecycles are also provided. See Table 1 for risk factors.

## 2. Biopsychosocial Risk Factors within the Perinatal Period Literature Review

### 2.1. Biological Risk Factors

During the perinatal period, women face a number of biological implications [1,4,5]. Further, it is the biological realm that receives the most attention, especially during gestation [1,5]. While we generally focus on physical and nutritional components of health (and will briefly discuss them here), we expand our discussion to include neurobiological issues, genetic influences, and adverse childhood events (ACEs).

#### 2.1.1. Physical Concerns

Pregnancy involves a host of physical changes to a women’s body including hormonal changes (e.g., estrogen and progesterone release, weight gain, fluid retention, breast enlargement); sensory changes (e.g., change is smell and taste); hair, skin, and nail changes (e.g., brittle nails, hair loss, stretch marks); circulatory system changes (e.g., blood pressure, dizziness, fainting); and respiratory and metabolic changes (e.g., body temperature, metabolic rate, dehydration) [6,7]. Medications are included under physical concerns, as some women are severely limited in the medications that they can consume when they are pregnant. However, advising women to discontinue medications presents risks such as untreated mental illness, nutritional concerns, poor adherence to care, and increased tobacco and alcohol intake [8]. Further, mental health-related medications are almost never suggested during pregnancy and can therefore place the mother at risk of negative perinatal and postnatal outcomes [8]. Additionally, Lockwood and Youssef conducted a systematic review of epigenetic effects of pharmacological agents and found “epigenetic changes associated with the use of mood stabilizers or antipsychotic medications” [9] (p. 1). Therefore, it is essential to consider the maternal and neonatal impacts of medications (or lack thereof) on the physical aspects of health in the pregnancy lifecycle.

#### 2.1.2. Nutritional Concerns

Poor nutrition is a factor contributing to healthcare issues in women and their children [10]. Vitamin deficiencies associated with nutritional concerns often correlate with poverty, diseases, and poor food accessibility [10,11,12]. As sufficient nutrient intake is a critical component of a healthy pregnancy outcome, women in their perinatal periods are at risk of concerns influencing their personal wellbeing and the wellbeing of their newborns. Hanson et al. [10] conducted a study on 108 women (in the United States) at the time of delivery and found that race and insurance status were associated with vitamin A and E deficiencies and that non-white women were at risk for deficiencies at higher rates than their white counterparts. Thus, women in wealthier countries (such as the U.S.) can experience deleterious effects having a lack of access to healthy food options during the perinatal period.

#### 2.1.3. Neurobiological Concerns

According to Hoekzema and colleagues [13], pregnancy leads to changes in women’s brain structure. Specifically, pregnancy impacts the amount of gray matter in the brain and influences areas related to social cognition, primarily in the cerebral cortex [13]. These changes in the neurobiology of women’s brains are long-lasting and could influence social processes like enhancing a mother’s abilities to recognize emotional needs [14,15]. Further, it could be speculated that maternal neurobiological changes could directly influence parent–neonatal attachment, as a mother’s ability to appropriately respond to a newborn’s needs is integral in neonatal mortality and basic parent–neonatal bonding [16]. An additional neurobiological concept in the perinatal period is that of epigenetics, or genetic modifications induced by environmental factors that impact the expression of genes [17]. Predisposition to health and illness is in part based off the genetic make-up of the child’s parent [18]. According to Bale [19], genetic susceptibility to mental and physical illness has three components: (1) genetic predisposition, (2) prenatal stress, and (3) exposure to stress after birth. For the purposes of this review, it is important to emphasize the second component, the stress of the mother during pregnancy as a major factor in creating genetic vulnerability to illness in their unborn child. Specifically, as the work of Buss and colleagues presents [20], stress during pregnancy can have significant impacts on the development of organ systems and brain structures. Furthermore, the stress of the mother can have a large impact on the stress response system of the fetus throughout its lifespan [21]. This aspect of the biopsychosocial model is important for all mothers, but especially those who identify as WOC and experience chronic stress through their lifespan and throughout their perinatal experience. One aspect of lifespan stress that has been well documented is the experience of adverse childhood experiences.

#### 2.1.4. Adverse Childhood Events (ACE)

Adverse Childhood Experiences (ACE) involve negative/traumatic experiences experienced as a child (prior to the age of 18). These ACEs are increasingly being linked to adverse perinatal outcomes, in particular, there is emerging evidence that increased ACEs burden in a diverse low-income population can predispose women to increased pregnancy loss, low-birthweight, and premature delivery [22]. Specifically, cumulative ACE scores are associated with increased incidence of each of these untoward outcomes [22]. Understanding the ACE exposure of a maternal population from a mental health perspective can also allow for a trauma informed approach to care. In a meta-analysis evaluating risk of anxiety and/or depression, Racine et al. [23] describe a small to moderate impact on maternal mental health from ACEs. Routine screening for ACEs is recommended in order to properly assess risk for poor pregnancy outcomes [24].

### 2.2. Psychological Risk Factors

Pregnancy is often viewed as a psychological event in and of itself, with a number of complex changes occurring during the perinatal period [25]. According to Bjelica and Kapor-Stanulovic [25], women experience a range of changes such as mood changes, anxiety, fatigue, depression, exhaustion, and excitement. Further, the gestation period (e.g., pregnancy) can serve as a stressful event influencing both maternal and neonatal functioning [25]. Related to perinatal mental health specifically, Yehudah and Lorelle [5] discussed Black birth outcomes and stated that Black women are at an increased risk for poor long-term health outcomes. In the following section we highlight peri-partum and post-partum factors, stressors, and trauma experiences as a few of the psychological implications women face in this unique period.

#### 2.2.1. Peri-Partum Factors

The term peri-partum generally refers to the last month of gestation or the first few months after delivery. Peri-partum risk factors may include increased anxiety associated with the demands of parenthood, fatigue and exhaustion, body changes and a feeling of uncomfortableness, mood changes, and excitement and contribute to enhanced feelings of baby blues or more severe issues like Post-Partum Depression (PPD) [14,16,18,25]. In the psychological realm, additional peri-partum issues such as healthcare pressure, substance abuse issues, and a lack of communication with healthcare providers or pressure to make certain medical decisions, could increase pregnancy life cycle risks [25]. In a qualitative investigation, Jou et al. [26] found roughly 15% of women received pressure to follow certain birth plans (e.g., labor induction, cesarean delivery) and determined patient-perceived pressure predicted labor plans and that communication between patient-provider could maximize women’s right to choosing their birth plans/outcomes. In another metasummary investigation of N = 23 qualitative studies, Renbarger et al. [27] found that women in their pregnancy lifecycles with substance use disorders experienced their healthcare experiences as conflictual. Participants experienced providers as judgmental, scrutinizing, disempowering, deficient, and disparaging in nature [27]. Again, patient–provider communication was listed as an important variable in mitigating healthcare disparities. Building on communication, Nicholls et al. [28] utilized an interpretive qualitative methodology and found women in their perinatal periods could benefit from an empowering approach, where they feel like they are able to make choices about their bodies and their pregnancies. Further, a woman-first healthcare initiative could be advantageous, as opposed to the usual compliance culture within healthcare settings [28].

#### 2.2.2. Post-Partum Factors

Along with poor birth outcomes and risk-factors relating to the perinatal part of a pregnancy lifecycle, women face a variety of mental health issues later in their pregnancy experience. Baby blues, or acute Postpartum symptoms such as difficulty sleeping, mood changes, and difficulty concentrating are found in 3 out of 4 women post-gestation [29]. Postpartum Depression (PPD), which is a more extreme form of post-gestation issues, is classified as a mental health disorder, affects approximately 13% to 20% of women [29]. Further, Beck [30] found risk factors of PPD as: peri-natal pressures, prenatal depression, self-esteem, childcare stress, prenatal anxiety, life stress, social support, marital relationship, history of previous depression, neonatal temperament, maternity blues, marital status, SES, and unplanned/unwanted pregnancy. Many of the aforementioned risk factors involve areas of the life cycle of the pregnancy that are prior to giving birth and/or post-gestation. As such, learning about the experiences and issues occurring for women prior to gestation and during, should allow helping professionals to intervene earlier and prevent negative pregnancy lifespan issues/outcomes. One of the primary risk factors for many pre- and post-partum psychological distress is the presence of stress.

#### 2.2.3. Stressors

Mental wellness is an important factor for women in perinatal periods. As introduced in the neurobiological section, pre-and-post-natal stressors are associated with biological and physical concerns (e.g., mental and physical illness) [19]. Furthermore, issues like anxiety and stress during pregnancy lifecycles are associated with a number of negative maternal and neonatal outcomes such as spontaneous abortion, preterm delivery, and delivery complications [8]. Mortality rates among WOC are substantially higher for Black women (10.97 for every 1000 live births), than for their White and Hispanic counterparts [31]. These high mortality rates for Black women are linked with stress related pregnancy complications [31]. Further, Black women have higher chance of delivering their children early, and once delivered their children have a higher chance of weight loss or low birth weight—effects associated with maternal stress during the early stages of the pregnancy life cycle [31,32,33]. All women are at risk of experiencing stress-related concerns during their perinatal period of pregnancy [19].

Holzman and colleagues [34] use the term weathering phenomenon to describe this acute and accumulative stress experience. This term aligns with the counseling/mental health field’s concept of intergenerational or historical trauma and wellness across the lifespan [33,35]. The weathering phenomenon are the individual or environmental conditions that mothers endure across generations or their lifespan, causing acute and chronic stress or physical symptoms such as inflammation and a higher risk for infections [32]. These conditions increase the likelihood of preterm birth and other adverse health conditions across the child’s lifespan such as obesity and hypertension [36,37]. Another term associated with experiencing acute and chronic stress is transgenerational trauma.

#### 2.2.4. Transgenerational Trauma

Transgenerational trauma refers to experiences that pass-through generations, with the basis that individuals can experience trauma and pass symptoms and behaviors of trauma survival on to their offspring [38]. Besides the influence of trauma on psychological processes (e.g., mental wellness of those exposed), transgenerational trauma can “affect the biology of the individuals, and even have biological and behavioral consequences on the offspring of exposed individuals” [38] (p. 1). As such, this concept is relevant to women in their perinatal periods of pregnancy, considering that past traumas and experiences can influence present and future levels of functioning. When referring to communities of color, it is not only WOCs current socio-economic experiences that increase their vulnerability to adverse birth outcomes (e.g., generational poverty), but also the historical socio-economic experiences at play [39]. In the U.S., examples of historical trauma include slavery, gendered racism, sexual trauma, and discriminatory healthcare practices [40]. These issues are experienced disproportionately by WOC (especially Black women) and increase the chances for offspring experiencing and “living” the trauma of their mothers [40].

### 2.3. Sociological Risk Factors

As with biological and psychological implications to maternal and neonatal health, social determinates are at play as well. A social implication in the pregnancy lifecycle may include SES, discrimination/race-based issues, love and friend relationships, and overall access to quality healthcare services.

#### 2.3.1. Income/Poverty

The link between poverty and maternal wellbeing has been established, with lower-income women experiencing risk-factors negatively impacting maternal and neonatal outcomes [41]. For example, women in poverty experience increased malnutrition, higher drug and alcohol usage, and increased psychological stressors [42,43,44,45]. Further, lower income mothers have less access to family planning services and lower incomes are correlated with child health and wellbeing [46]. Hamad and Rehkopf [47] found associations between income and neonatal birthweight, with increased birthweight tied to higher financial means. In addition to income concerns for women, higher income is associated with lower pre-term births for White women, but not for WOC [48]. Cubbin and colleagues [49] stated that poverty and income-inequality were associated with adverse birth outcomes for WOC and discrepancies due to inequities in healthcare experiences were persistent despite medical and prevention efforts.

#### 2.3.2. Discrimination

For women in their pregnancy lifecycles, discrimination is a topic that has been researched heavily in the last few years [50,51]. Nearly one-fifth (roughly 20%) of women report experiences of discrimination by health care providers during prenatal care, labor, or delivery [52]. Further, pathogenic factors linked to race continue to affect health, even when controlling for SES. For example, racial disparities in health, including self-rated health, heart disease mortality, hypertension, and obesity exist at every level of SES [53]. Regarding education, Black women with a college degree or higher have a higher rate of neonatal mortality than White, Latinx, and Asian and Pacific Islander women who have not completed high school [53]. Consequently, these results suggest additional factors linked to racial status exist, adding to healthcare disparities for WOC during the perinatal period. The racial disparities evident in the medical health system not only have physical impacts on women but highlight a need for healthcare providers to better understand how these disparities might impact women’s mental health and wellness, as both are important in maternal and neonatal health outcomes [49]. Women of Color are especially at risk of experiencing disparities when navigating the current healthcare system in the U.S.

#### 2.3.3. Race

Along with discrimination, racial disparities in birth outcomes are longstanding and well-documented in public health data and epidemiological literature [54,55]. Regarding maternal and neonatal health, Black women and Hispanic women are three to four times more likely to die from childbirth than White women [56]. Further, Black women are nearly three times as likely to have a child die within one year of birth than Hispanic women and White women [56]. Because of the disparate rates of adverse pregnancy lifespan outcomes for WOC, it is important to discuss perinatal experiences and to focus on variables contributing to inequalities and the impact on health and wellness.

Vedam et al. [57] surveyed N = 2138 women and concluded that 1 in 6 women reported experiencing one or more types of mistreatments (as measured by their Giving Voice to Mothers; GVtM scale), and that WOC experienced mistreatment most frequently. Further, mistreatment during the pregnancy lifecycle was higher for births occurring in hospitals and among participants experiencing economic, health, or social challenges [57]. Finally, women who experienced unexpected events (e.g., unplanned cesarean) and women who disagreed about the treatment plan for themselves or for their offspring, experienced the highest rates of mistreatment [57]. The most common form of mistreatment was verbal based (e.g., failure to respond to requests for help) with protective factors of: identifying as White, having a vaginal birth, having a baby after 30 years of age, and giving birth outside of the hospital [57]. Similarly, McLemore et al. [58] assessed perinatal healthcare experiences of women (N = 54) and found participants experienced: (a) disrespect during healthcare encounters (e.g., discrimination and racism), (b) stressful interactions, (c) unmet information needs, and (d) inconsistent social support, with WOC describing increased disrespect, inconsistent social support, and stressful interactions more frequently. However, enhanced provider-patient communication might mitigate negative healthcare experiences for women and help foster a person first (e.g., woman first) healthcare narrative [58].

#### 2.3.4. Relational Aspects

Women in their perinatal periods receive a different level of relational support. This provision could be positive, in the form of partner support and enhanced love and friend relationships. Regarding partner support, women who perceive social support from their partners have lower postpartum stress [59]. Regarding relationships, satisfaction related to relationship qualities (e.g., intimacy) has also been related to postpartum mental wellness [60]. Friends may also offer additional social support and foster a sense of maternal security throughout the perinatal period [59]. Relational aspects for women during pregnancy can also be negative, such as experiencing intimate partner violence (IPV) [61], which can negatively influence maternal and neonatal health. Risk factors for women to experience IPV during their perinatal period include young age, single relationship status, minority racial and ethnicity status, low SES, and rates of abuse [62,63]. Though relational aspects can be beneficial to women in the perinatal period, a lack of social support can have direct negative implications for women and their future offspring.

#### 2.3.5. Access to Insurance/Quality Healthcare Services

Access to health insurance is considered a step in receiving quality healthcare [56]. With the expansion of healthcare in the U.S. through avenues such as the Affordable Care Act (ACA), roughly 20 million more people received access to health insurance [56] and recent research supports the idea that access to health insurance supports maternal and neonatal health and wellness. For example, Bhatt and Beck-Sagué [64] investigated the influence of a Medicaid expansion in the ACA and found that between 2014 and 2016, neonatal mortality rates fell in states that expanded Medicaid and rose in states that did not expand access to the program. Along with insurance, access to affordable reproductive care is also a risk factor for women in their perinatal periods. Many women (especially WOC) do not have access to reproductive healthcare and consequently have less options for family planning [46]. The ability to plan a pregnancy is associated with better health outcomes and affords women more control during the perinatal period), often reducing the risk of pregnancy-related complications [56,65]. The aforementioned section highlighted biopsychosocial risk factors in the perinatal pregnancy lifecycle. Now, implications on reducing health disparities associated with these risks are presented.

## 3. Discussion

Though a number of risks exist for women in perinatal periods, there are a number of protective factors. Protective factors for women in the perinatal period include: (a) increased access to health insurance, (b) access to quality health care, (c) access to providers that practice patient-centered and culturally sensitive care, and (d) access to reproductive healthcare (contraception, abortion, sexually transmitted infections screenings, reproductive cancer screenings) [56]. Prather and colleagues [40] suggested that healthcare professionals: focus on culturally and contextually appropriate research and prevention, provide equal access to sexual and reproductive healthcare information and services, provide quality education and training for helping professionals, and support policies for positive sexual and reproductive women’s healthcare. We echo these sentiments and suggest while working with women during their pregnancy lifecycle, healthcare professionals utilize a biopsychosocial clinical model, addressing the holistic needs of their patients. Using a biopsychosocial model involves collaboration with multiple stakeholders contributing to the patient’s health and may incorporate non-traditional medical services such as doula services and/or adding an in-home component which could be especially helpful for WOC [66]. Additionally, healthcare professionals can support perinatal wellness by providing culturally responsive services, advocating for their patients, and allowing patients to be assertive regarding their medical needs.

### 3.1. Implications for Healthcare Professionals

#### 3.1.1. Culturally Responsive Services

Healthcare professionals are trained to provide multiculturally competent services, meeting patients where they are in empathic and genuine ways [67]. Since women’s healthcare is a biopsychosocial issue, a wellness philosophy is a fit for this population, as it allows for a holistic examination of factors influencing human functioning. Furthermore, recognizing gendered racism, depression, anxiety, and stress, could promote enhanced maternal health outcomes [5,68]. In addition, utilizing appropriate models based on women’s needs (e.g., operating from racial-cultural competency and utilizing race broaching skills when necessary) could be beneficial when working with diverse patients.

#### 3.1.2. Advocacy Role

Factors such as medication/non-medication management and nutritional concerns in the biological realm, peri-and-post-partum issues in the psychological realm, and societal issues such as racism and socioeconomic influences in the social realm are paramount in providing sound clinical treatment for women in pregnancy lifecycles. As there are many variables at play, healthcare professionals might benefit women by taking a supportive role and aiding patients through advocacy work in medical settings. It may also behoove healthcare professionals to work in tandem with other helping professionals in an interdisciplinary nature [5]. When having conversations with women, it is especially important for counselors to consider and discuss social influences such as racism, microaggressions, race broaching, and cultural factors that could be influencing perinatal wellness [69]. Taking it a step further, health professionals can advocate and act against systemic power barriers affecting marginalized patients and take steps to advocate for equitable access to quality healthcare, reasonable insurance plans, and culturally-sound healthcare providers [69].

#### 3.1.3. Patient Assertiveness

If women are empowered to “ask for” what they need/want and take an assertive stance in their healthcare, negative outcomes related to “not hearing women” and not trusting women as the experts over their own bodies, might be mitigated. Ultimately, doctors, health providers, and other helpers who are not meeting the individual patient’s needs can be replaced with individuals who listen and take into account the unique biopsychosocial variables influencing perinatal health.

## 4. Limitations of the Literature Review

As with any review, there are limitations. This thematic review focused on perinatal risk factors by reviewing literature associated with biological, psychological, and social determinants of health. Though many factors were discovered and discussed, the review is not exhaustive, as there may be additional risk to women in their perinatal period not included in this review. Furthermore, this paper includes a general review of the available literature, and the information and conclusions may not be applicable to all perinatal women and their neonatal offspring.

## 5. Conclusions

Women face risks to their perinatal wellbeing and it benefits healthcare professionals to have knowledge of the biopsychosocial determinants of health. Knowing the risks within a biopsychosocial paradigm allows for accountability and enhanced quality of care for expectant mothers. Incorporating and supporting protective factors for women in their perinatal period includes increasing access to health insurance and quality health care, providing patient-centered and culturally sensitive care, and enhancing access to reproductive healthcare. Further, healthcare professionals can support perinatal health by using this holistic model to assess for potential risks and discuss protective factors, while viewing each patient from their unique, diverse background. Ultimately, utilizing a biopsychosocial model can enhance discussions on the numerous facets influencing the perinatal period and increase patient/healthcare professional communication.

## Figures and Tables

**Table 1:** Biopsychosocial model of wellness: risk factors for women in pregnancy lifecycles.

Tenets	Risk Factors	Components
**Biological**		
	Physical	Body changes, hormonal changes, medication concerns, epigenetic changes
	Nutritional	Poor nutrition, vitamin deficiencies, lack of access to quality food
	Neurobiological	Neurological changes (e.g., brain structure changes), genetic predisposition to illness/health
	Adverse Childhood Events (ACEs)	Negative/traumatic experiences of childhood
**Psychological**		
	Peri-Partum	Anxiety, fatigue/exhaustion, baby blues, mood changes (in last month of gestation or first few months after delivery)
	Post-Partum	Baby blues, post-partum depression (PPD), depression, self-esteem issues, anxiety, stress, lack of support (in post-gestation)
	Stressors	Caused by biological components (e.g., physical changes, nutritional demands, neurobiological changes, traumatic events), mental wellness concerns, anxiety, lack of support, weathering phenomenon
	Transgenerational Trauma	Past traumatic experiences influencing offspring
**Sociological**		
	Income/Poverty	Lower SES associated with negative maternal and neonatal outcomes
	Discrimination	Unjust treatment, negative impacts on maternal and neonatal outcomes
	Race	Racial disparities in birth outcomes, most common form is verbal-based discrimination, WOC experience worse maternal and neonatal pregnancy lifecycle outcomes
	Relational Aspects	Love, friendship, social support, intimate partner violence (IPV)
	Access to Insurance/Quality Healthcare	Insurance plans, family planning access/reproductive care
